# A serological survey of human adenovirus serotype 2 and 5 circulating pediatric populations in Changchun, China, 2011

**DOI:** 10.1186/1743-422X-9-287

**Published:** 2012-11-23

**Authors:** Bin Yu, Zhen Wang, Jianing Dong, Chu Wang, Lina Gu, Caijun Sun, Wei Kong, Xianghui Yu

**Affiliations:** 1 National Engineering Laboratory for AIDS Vaccine, College of Life Science, Jilin University, Changchun, 130012, China; 2 Key Laboratory for Molecular Enzymology and Engineering, the Ministry of Education, College of Life Science, Jilin University, Changchun, 130012, China; 3 Department of ICU, The First Hospital of Jilin University, Jilin University, Changchun, 130021, China; 4 State Key Laboratory of Respiratory Diseases, Center for Infection & Immunity, Guangzhou Institute of Biomedicine and Health, Chinese Academy of Sciences, Guangzhou, 510530, China

**Keywords:** Adenovirus, Seroprevalence, Neutralizing antibodies, HIV vaccine

## Abstract

**Background:**

Efficacy of recombinant adenovirus serotype 5 (rAd5) vaccine vectors for human immunodeficiency virus type 1 (HIV-1) and other pathogens have been shown to be limited by high titers of pre-existing Ad5 neutralizing antibodies (NAbs) in the developing world.

**Results:**

Using a secreted embryonic alkaline phosphatase (SEAP) neutralization assay, 50% serum neutralization titers against rAd2 and rAd5 vectors were measured in samples from 274 infants and young children in northeast China. The pediatric population was found to be 59.6% and 43.3% seropositive for rAd2 and rAd5, respectively. Of all participants, 44.9% had moderate and high (> 200) and 25.6% had high (>1000) Ad2 NAb titers, compared with the corresponding rates of 26.6% and 9.3% against Ad5. Marked age-dependent increases in NAb titers to both Ad serotypes were observed across five age groups, with the exception of infants in the 0-6-month group commonly having relatively high titers due to pre-existing maternal antibodies.

**Conclusions:**

Our data suggest that Ad-based therapies may be suitible for children in the 7-12-month age range in this region.

## Background

Adenoviruses (Ad) are non-enveloped, double-stranded DNA viruses of the *Adenoviridae* family which infect a wide range of vertebrate hosts. There are over 50 different serotypes of Ads of the genus *Mastadenovirus* known to infect humans and are divided into six subgroups, A to F [1]. Adenoviral vectors have been used for a variety of vaccine applications against various cancers and infectious diseases. Unfortunately, a majority of the human population has been infected by wild-type Ads in childhood, and often these individuals harbor pre-existing immunity to a number of common Ad serotypes (i.e., serotypes 2, 4, 5 and 7) [2,3]. The clinical application of these Ad serotypes in construction of vaccines may be seriously hampered due to neutralizing antibodies (NAbs) to these vectors [4]. Baseline levels of Ad5 NAbs have been shown to suppress the immunogenicity of rAd5 vector-based vaccines for HIV-1 both in preclinical studies [5] and in clinical trials [6].

The seroprevalence of Ad serotypes commonly used in vaccine clinical trials, such as Ad2 and Ad5 from subgroup C, were found to be higher than other types in Europe, Asia and the United States [7]. An international epidemiological study on Ad type-5, -6, -26 and −36 showed that the overall proportion of anti-Ad5 seropositive individuals was 85.2%, and Ad5 NAb titers varied by geographic location, appearing to be higher in non-US and non-European settings [8]. In that study, Asian (Thai) participants had the highest Ad5 NAb titers; however, samples from China were not included. Meanwhile, one report indicated that Ad5 NAb titers were low in young children in sub-Saharan Africa [9]. Appaiahgari *et al.* showed in an experimental mouse model that low levels of Ad5 NAbs had no effect on the protective efficacy of an Ad5-derived recombinant virus expressing the Japanese encephalitis virus envelope protein [10]. These observations raise the possibility of using Ad5-derived recombinant vaccines for immunization in populations with low Ad5 NAb levels.

A recent study of Ad5 in Guangzhou (capital city of Guangdong province in southern China) demonstrated that 77.4% of the 200 subjects evaluated were seropositive, showing an intermediate prevalence of Ad5 infection in southern China [11]. However, our prior epidemiological study of Ad5 NAbs showed that areas in southern China may have a higher seroprevalence of Ad5 than in other regions [12]. The objective of this current study was to determine the prevalence of NAbs against both Ad2 and Ad5 in pediatric populations in northeast China. The serum samples of children from 1 day to 12 years of age were divided into different age groups to monitor their antibody titers. The finding of age-dependent Ad NAb titers in this study may have implications for the clinical application of Ad-based vaccines and gene therapy in China.

## Materials and methods

### Human serum samples

A total of 274 serum samples were obtained from healthy children from 1 day to 12 years of age, living in Changchun city, Jilin (province in northeast China). All of the samples were collected randomly from February to October in 2011. Informed consent was obtained from the legal guardians of all participants, and the serum samples utilized in Ad neutralization assays were approved by the Human Ethical Committee Institutional Review Board of Jilin University, Jilin, China. All human sera were inactivated at 56°C for 60 min prior to testing.

### Virus neutralization assays

The Ad2-SEAP and Ad5-SEAP vector was constructed as previously described [7,13]. In these E3 deletion vectors, the E1 region was replaced by the SEAP gene. Ad-specific NAb titers were assessed using a secreted embryonic alkaline phosphatase (SEAP) quantitation assay [14]. Briefly, HEK293 cells were infected with Ad2-SEAP or Ad5-SEAP. Concurrently, 4 × 10^6^ viral particles (vp) of Ad-SEAP were incubated for 1 h at 37°C either alone (set as 100% SEAP activity) or with five serial dilutions of serum in DMEM with 10% fetal bovine serum (FBS) before infection. The final serum dilutions were 1:18, 1:72, 1:288, 1:1152 and 1:4608, and each serum sample was tested in duplicate. After incubation at 37°C for 24 h, supernatants were collected and assayed for SEAP activity using the chemiluminescent substrate CSPD (Roche, Basel, Switzerland) according to the manufacturer’s instructions. Luminescence intensity was measured using a Victor 1420 Multilabel counter (Perkin Elmer, Wellesley, MA, USA). The neutralization titer was defined as the inverse of the serum dilution at which SEAP activity was reduced by 50% of that with the virus alone.

### Statistical analysis

Logistic regression was used to calculate the odds ratios (OR) and 95% confidence intervals (CI) to analyze factors associated with moderate and high Ad2 and Ad5 NAb titers (>200) in all samples. A binary variable was established to represent Ad NAb titers >200 or low/negative titers ≤200 among each group of samples. Univariate logistic regression analysis identified variables associated with moderate and high NAb titers at the *P* < 0.05 level of significance. Standard deviation and/or percentages were analyzed by SPSS 16.0 using Chi-square and t tests.

## Results

**Figure 1:**
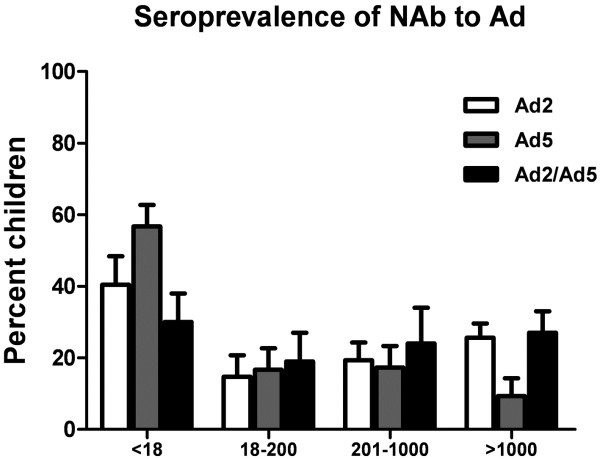
Ad2, Ad5 and Ad2/Ad5 (Ad2 + Ad5) NAb titers in serum samples of all children stratified by titers: <18 (negative), 18–200 (low), 201–1000 (moderate) and >1000 (high)

**Figure 2:**
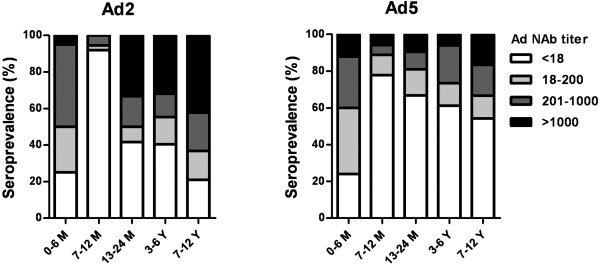
**Seroprevalence of Ad2 and Ad5 in different age groups.** Number of serum samples in each age group: 50 (0–6 M), 37 (7–12 M), 42 (13–24 M), 97 (3–6 Y), and 48 (7–12 Y). M = months; Y = years

**Figure 3:**
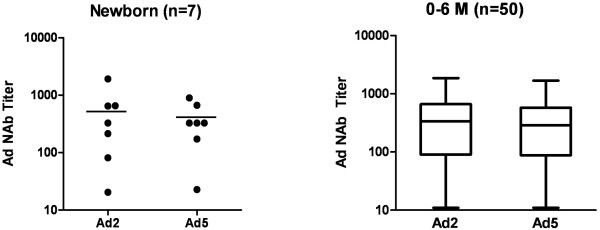
Ad2 and Ad5 NAb titers in serum samples from 7 individual newborn infants (< 3 days) (bars, median titers) and 50 0-6-month-old infants (box-and-whisker plots)

We first evaluated the prevalence of NAbs against Ad2-SEAP and Ad5-SEAP in sera taken from 274 children from 1 day to 12 years of age. To determine the relative differences in titers in different populations, Ad NAb titers were divided into subgroups, similar to those reported for other Ad seroprevalence studies [15]. As shown in Figure 1, Ad2 and Ad5 seroprevalence rates were 59.6% and 43.3% in all test populations, respectively. It was found that 44.9% of participants had moderate and high Ad2 NAb titers (> 200) and 25.6% had very high titers (> 1000), compared with the corresponding rates of 26.6% and 9.3% against Ad5. The results also showed that over 60% of children in northeast China were positive for NAbs (titer > 18) against either the Ad2 or Ad5 serotype, and 51.0% had moderate and high levels of Ad2/Ad5 NAb titers. To evaluate the prevalence of neutralizing antibodies against Ad2 and Ad5 in infants (0–24 months old) and children (3–12 years old), frequency histograms were drawn to illustrate the impact of age on Ad2 and Ad5 infection. Serum NAb levels are shown for five separate age groups in Figure 2. It was found that the percentages of individuals seronegative to both Ad serotypes increased with increasing age, with the exception of the 0-6-month-old group showing a larger portion of low (18–200) and moderate (201–1000) titers than other groups. In this youngest group, 74.6% and 77.2% were seropositive to Ad2 and Ad5 NAbs, respectively. It also appeared that the 7-12-month-old group had the lowest percentages (Ad2, 5.4%; Ad5, 11.2%) of baseline Ad NAb titers, and the percentages of baseline titers increased with increasing age from this age group up to the 7-12-year-old group. Additional interesting findings were that serum samples in over 50% of the 7 newborn infant (<3 days) had baseline Ad2 and Ad5 NAb titers, and the median Ad2 and Ad5 NAb titers were 368 and 288 in the 0-6-month-old group, respectively (Figure 3). 

Univariate analyses of factors associated with moderate and high Ad2 and Ad5 titers are shown in Table 1. Unadjusted regression suggested a trend where moderate and high Ad2 titers increased along with age when the children over the age of 12 months. Ad5 titers displayed a similar valley-like trend, and these associations were weak and not statistically significant. In addition, no apparent differences in Ad2 or Ad5 seroprevalence between genders were found in all pediatric populations.

**Table 1:** Unadjusted and adjusted odds of moderate and high Ad2 and Ad5 NAb titers in children of northeast China

**Population**	**Ad2 NAb Titers >200**			**Ad5 NAb Titers >200**		
	**No. (%**^**a**^**, %**^**b**^**)**	**Unadjusted OR (95% CI)**	**Adjusted OR (95% CI)**	**No. (%, %)**	**Unadjusted OR (95% CI)**	**Adjusted OR (95% CI)**
Overall	123 (44.89)			74 (27.01)		
Age						
0-6 mo	25 (50.00, 20.33)	1.00		20 (40.00, 27.03)	1.00	
7-12 mo	2 (5.41, 1.63)	0.06 (0.01, 0.26)	0.07 (0.02, 0.30)	4 (10.81, 5.40)	0.18 (0.06, 0.59)	0.33 (0.11, 0.96)
13-24 mo	21 (50.00, 17.07)	1.00 (0.44, 2.27)	1.23 (0.64, 2.35)	8 (19.05, 10.81)	0.35 (0.14, 0.92)	0.64 (0.28, 1.44)
3-6 yr	45 (46.39, 36.58)	0.87 (0.44, 1.71)	1.06 (0.67, 1.69)	26 (26.80, 35.14)	0.55 (0.27, 1.13)	0.99 (0.59, 1.67)
7-12 yr	30 (62.50, 24.39)	1.67 (0.74, 3.73)	2.05 (1.09, 3. 85)	16 (33.33, 21.62)	0.75 (0.33, 1.71)	1.35 (0.70, 2.61)
Sex						
Male	58 (39.73, 47.15)	1.00		40 (27.40, 54.05)	1.00	
Female	65 (50.78, 52.85)	1.56 (0.97, 2.53)		34 (26.56, 45.95)	0.96 (0.56, 1.64)	

OR = odds ratio; CI = confidence interval. mo = months; yr = years.

^a^ Data as a percentage of overall samples in each age or sex group.

^b^ Data as a percentage of overall data (Ad NAb titers >200).

## Discussion

The high prevalence of human anti-Ad5 Abs, especially in sub-Saharan Africa, suggests that administration of Ad5-based vectors will likely lead both to antibody neutralization and a rapid, exacerbated cellular immune response in many individuals [16]. The practical scope of this problem was illustrated by the HIV-STEP trial, the first clinical trial aimed towards eliciting vaccine-induced cellular HIV immunity in humans. In participants with high initial anti-Ad5 antibody titers, the vaccine appeared to have increased HIV infection [17]. As HIV vaccine trials are now underway in children [18], it is important to determine at what age Ad NAbs become more prevalent. However, few studies on the seroprevalence of NAbs to Ad5 or other serotypes in pediatric populations, especially infants, have been published to date. Previous studies indicated that Ad5 NAb titers are low in young children, and immunity to Ad5 vector is age-dependent [9,14]. However, a recent international epidemiological study of several Ad types reported a high Ad5 seroprevalence (65.9%) and correspondingly high-titer NAbs (titers > 1000) (43.2%) in children of South Africa [19]. In China, only one prior study in Guangdong demonstrated that 77.9% (28/36) of the participants were seropositive for Ad5 NAbs [11]. Our data showed that the seroprevalence of Ad5 NAbs in northeast China was apparently lower than that that in southern China, especially for the high-titer NAbs (18.8% vs. 30.6%), indicating that the anti-Ad5 titers may vary by geographic location.

In all 6 subgroups of Ad serotypes, Ad1, 2, 5 and 6 from subgroup C have been demonstrated to be more seroprevalent than serotypes in other subgroups [7,8]. Our current observations of the seroprevalence of Ad2 NAbs in China confirmed and extended the findings of prior studies. Pre-existing immunity to Ad can include both neutralizing antibodies, as well as Ad-specific T cell responses, as recent studies have highlighted a critical role for CD8^+^ T cells in pre-existing anti-Ad immunity [20-22]. It should also be noted that the magnitude and phenotype of cellular immune responses elicited by Ads may be cross-reactive. Considering the possibility of cross-reactivity within the same subgroup which may lead to a high seroprevalence of Ad even in infants or young children, our analysis of Ad2/5 NAb titers suggests that the approach of combining different Ad vaccine vectors for heterologous priming or constructing chimeric Ad vectors to overcome pre-existing immunity will need to be carefully designed.

High titer of Ad5 NAbs have been shown to suppress the immunogenicity of rAd5 vector-based vaccines for HIV-1 in both preclinical studies [5,23] and clinical trials [6,24,25]. In this study, significant differences in Ad antibody titers were monitored by performing univariate analysis of the association of age with moderate and high Ad2 and Ad5 titers. The levels of moderate and high Ad2 and Ad5 NAbs in children showed an age-dependent increase along with Ad-specific immunity, with 7-12-month-old infants having the lowest levels of anti-Ad immunity. We also found that samples from the youngest infants (0–6 months), even from seven newborn infants (< 3 days), had moderate and high titers of Ad2 and Ad5 NAbs. These results are consistent with prior studies showing that the pediatric population harbors anti-Ad titers at birth due to passively acquired maternal Abs which substantial decline by 6 months of age [8,9] but then drastically increase again after 18 months of age [26]. In the present study, NAbs for the Ad2 serotype were shown to increase markedly even after 12 months of age (Figure 2 and Table 1). These results indicate that adenovirus-based therapies may not be suitable for children above 12 months and below 6 months of age. These findings also suggest that before using Ad-based vaccines in a specific region, a thorough serological survey of NAbs for different Ad types in pediatric populations should be carried out, since pre-existing immunity may result in adverse inflammatory responses [19].

In summary, the seroepidemiological assessment conducted in this study revealed an interesting age-dependent association of anti-Ad2 and Ad5 NAbs in pediatric populations in northeast China, Thus, potentially high NAb titers to Ad in newborns or older children (>12 months) should be taken into consideration when designing Ad-based vaccines, while children of the 7-12-month age range with relatively low Ad-specific immuity may benefit the most from such vaccines.

## Abbreviations

rAd5: recombinant adenovirus serotype 5; NAbs: Neutralizing antibodies; HIV: Human immunodeficiency virus; SEAP: Secreted alkaline phosphatase; OR: Odds ratios; CI: Confidence intervals.

## Competing interests

The authors declare that they have no competing interest.

## Authors’ contributions

BY, ZW and JND performed virus neutralization assays. CW and CJS constructed the replication-incompetent rAd-SEAP vectors. LNG contributed to collection of specimens and clinical diagnosis. WK and XHY designed the study and critically revised the manuscript. All of the authors read and approved the final version of the manuscript.
